# Emergency Cricothyroidotomy for Acute Epiglottitis in a Patient With Super-Super Obesity: A Case Report

**DOI:** 10.7759/cureus.115584

**Published:** 2026-09-01

**Authors:** Alexander M Bhatt, Brandon Wang, Sung Hwa Dong, Ahmed Treki, Kay Lee

**Affiliations:** 1 Anesthesiology, Albert Einstein College of Medicine, New York City, USA

**Keywords:** cricothyroidotomy, difficult airway management, emergency surgical airway, epiglottitis, failed tracheal intubation, obstructive sleep apnea, super super obesity, videolaryngoscopy

## Abstract

Acute epiglottitis is uncommon in adults but can obstruct the airway within hours. Although few patients require airway intervention, those who do present one of the highest-risk intubation scenarios in clinical practice. Obesity further complicates physiological or technical challenges of airway management. It shortens safe apnea time, weakens the seal of a facemask or supraglottic device, hides the neck landmarks needed for a surgical airway, and can make imaging of the neck uninterpretable.

A 39-year-old man presented with two days of sore throat, dysphagia, and a muffled voice. He had asthma, hypertension, and severe obstructive sleep apnea treated with home continuous positive airway pressure. His body mass index was 66.5 kg/m^2^, with a weight of more than 200 kg. Urgent care had treated him for pharyngitis after a negative rapid streptococcal test. In the emergency department, the working diagnosis was angioedema. Intravenous dexamethasone and intramuscular epinephrine initially improved his symptoms over three hours, and then he deteriorated again. A lateral neck radiograph was reported as markedly limited by body habitus, and he declined a computed tomography scan because he could not tolerate supine positioning. After induction with intravenous ketamine, intubation with video laryngoscopy failed because the epiglottis was too swollen to admit an endotracheal tube, and oxygen saturation fell to 62%. Fiberoptic laryngoscopy was also unable to pass the swollen epiglottis, with a second desaturation to 69%. Laryngeal mask airway placement, only with an extensive two-handed jaw thrust maneuver, restored saturation to 99%, and a general surgeon performed a bedside cricothyroidotomy, placing a 6.0 mm endotracheal tube 40 minutes after the decision to intubate. Tracheostomy was attempted in the operating room but aborted because the trachea lay too deep. On hospital day 4, an otolaryngologist drained an epiglottic abscess and exchanged the cricothyroidotomy tube for an oral endotracheal tube through direct laryngoscopy with rigid and fiberoptic bronchoscopies, and cultures grew group G beta-hemolytic *Streptococcus*. Severe acute respiratory distress syndrome, biventricular systolic dysfunction, bilateral deep vein thromboses despite prophylaxis, and a peroneal compressive neuropathy followed. He went home on hospital day 46 with the cricothyroidotomy stoma still open.

Three lessons follow. First, an improved response to steroids and epinephrine in supraglottic obstruction can still be temporary and should not be read as a trend. Second, in a patient of this size, one failed intubation attempt can be all that separates a stable airway from a "cannot intubate, cannot oxygenate" emergency. And, third, a cricothyroidotomy tube in a deep neck is not a definitive airway and may not hold its position, so the plan for converting it should be established before placement.

## Introduction

Acute epiglottitis, or supraglottitis, is a cellulitis of the epiglottis and the surrounding supraglottic structures. It can progress to complete airway obstruction. Vaccination against *Haemophilus influenzae* type b moved the disease into adulthood, where reported annual incidence ranges from roughly two to three per 100,000, and mortality remains near 1% [1,2]. Adult mortality has fallen over the past three decades but has not disappeared, and deaths cluster among patients who obstruct before the airway is secured [3].

Most adults with epiglottitis never have their airway instrumented. A meta-analysis of 56 studies and 10,630 patients found a pooled airway intervention rate of 15.6%, falling to about 10% by the end of the study period, with tracheal intubation attempted in 10.2% [1]. The attempts that do occur are dangerous. The pooled failure rate was 4.2%, roughly one in 25 [1]. Adults also decompensate later than children, tolerating a given degree of narrowing for longer before they obstruct, which is how an unremarkable sore throat becomes an emergency [4].

Severe obesity complicates multiple aspects of difficult airway management. Low functional residual capacity and high oxygen consumption shorten safe apnea time, so desaturation after a failed intubation attempt takes seconds rather than minutes. Added pharyngeal and neck tissue and reduced chest wall compliance make face mask ventilation harder and supraglottic seal less reliable, and positioning for laryngoscopy is a logistical problem in itself [5,6]. The neck landmarks needed for front-of-neck access are often impalpable. In one direct comparison, anesthesiologists located the cricothyroid membrane by palpation in 39% of obese patients versus 71% of non-obese patients, and larger neck circumference predicted failure [7]. Super obesity is usually defined as a body mass index of 50 kg/m^2^ or more and super-super obesity as 60 kg/m^2^ or more.

When these two conditions occur together, each rescue step is both more likely to be needed and less likely to work. Published descriptions are few. We report an adult with a body mass index of 66.5 kg/m^2^ and acute epiglottitis in whom video laryngoscopy and flexible bronchoscopy both failed, a laryngeal mask airway restored oxygenation only with a two-handed jaw thrust, and a bedside cricothyroidotomy succeeded where a tracheostomy could not be completed. The emergency department recorded the sequence minute by minute, so we can describe the crisis with unusual precision. We also describe the full 46-day course, because the crisis turned out to be the start of the illness rather than the end of it.

This article was previously presented as a meeting abstract at the 79th PostGraduate Assembly in Anesthesiology on December 12, 2025.

## Case presentation

A 39-year-old man came to a community emergency department with two days of sore throat. Overnight, it had progressed to difficulty swallowing, a sensation that his throat was closing, and a muffled voice. Earlier that day, he had been seen at an urgent care center, where a rapid streptococcal antigen test was negative, and he was given a parenteral corticosteroid and oral azithromycin for pharyngitis. His family reported that he was worse by the time they got home.

His medical history included asthma, hypertension, generalized anxiety disorder, depression, and severe obstructive sleep apnea treated with home continuous positive airway pressure. A home sleep study several years earlier had shown an apnea-hypopnea index of approximately 50 events per hour, a nadir oxygen saturation in the mid-30s, and more than three hours below 89% saturation over a single night. His body mass index was 66.5 kg/m^2^ at a weight of more than 200 kg, which meets the usual threshold for super-super obesity.

**Figure 1:**
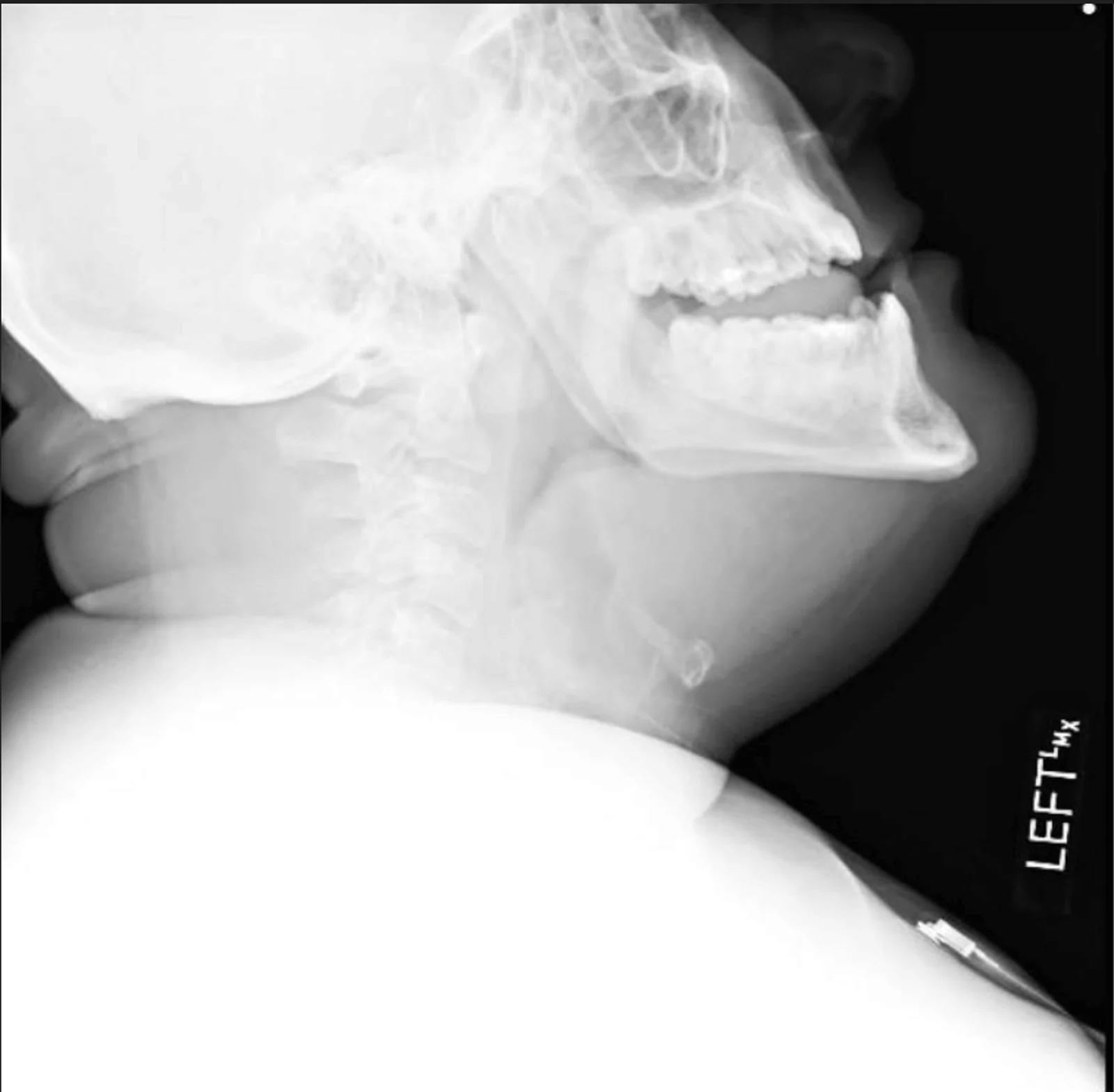
Lateral soft tissue neck radiograph at presentation Soft tissue edema is present in the submandibular region, but the radiologist reported the study as markedly limited by the patient's body habitus, with no further detail obtainable. The depth of the anterior cervical soft tissues, which accounts for the limited yield, is itself visible.

He was afebrile on arrival, with a heart rate of 155 beats per minute, a respiratory rate of 27, a blood pressure of 141/55 mmHg, and an oxygen saturation of 95% on room air. He was in distress and could communicate only by grunting and nodding. He had a muffled voice, mild inspiratory stridor, mild uvular edema, oropharyngeal and right tonsillar exudate, and a tongue his family reported was larger than usual. The white cell count was 16,250 per microliter with 90% neutrophils, C-reactive protein was 3.93 mg/dL, and lactate was normal at 1.3 mmol/L. Tests for group A *Streptococcus* and SARS-CoV-2 were negative. The chest radiograph was normal. A lateral neck radiograph showed submandibular soft tissue edema, but the radiologist reported the study as markedly limited by body habitus with no further detail obtainable (Figure 1).

The working diagnosis was angioedema. He received intravenous dexamethasone 10 mg, intramuscular epinephrine 0.3 mg, nebulized N-acetylcysteine and albuterol, intravenous ampicillin-sulbactam, and a liter of normal saline. He improved quickly. Within 40 minutes, the stridor and tongue swelling appeared to be settling; at one hour, he could manage short phrases, and by 90 minutes, he was speaking in full sentences. Computed tomography of the neck was ordered on the intensivist's advice, but about two hours later he declined it because he could not lie back on the scanner. Otolaryngology was consulted but was not immediately available.

Roughly four hours after the epinephrine, still speaking in full sentences, he reported feeling as though he were breathing through a straw. He became diaphoretic and tachycardic. Intubation was discussed with the patient and his family, including the possibility of a surgical airway, and consent was given. Table 1 shows what followed.

**Table 1:** Timeline of the airway crisis Times are elapsed minutes from the decision to intubate, derived from the contemporaneous emergency department record. Forty minutes passed between that decision and a secured airway, 27 minutes between the first laryngoscopy attempt and a secured airway, and eight minutes between skin incision and a secured surgical airway. SpO2: peripheral oxygen saturation; a dash indicates a value not recorded at that time.

Minutes	Event	SpO_2_
0	Decision to intubate, with consent obtained, including for a surgical airway if required	-
+2	Anesthesiologist at bedside	-
+4	Respiratory therapist at bedside	-
+9	Ketamine 300 mg IV	-
+12	Ketamine 100 mg IV	-
+13	Video laryngoscopy attempted; epiglottis too swollen to admit tracheal tube; bag-mask ventilation begun	62%
+17	Assisted bag-mask ventilation continued	90%
+22	Flexible bronchoscope at bedside, laryngeal mask airway placement attempted, general surgery called	-
+26	General surgeon at bedside	-
+28	Fiberoptic bronchoscope removed, laryngeal mask airway placed after desaturation	69%
+29	Local anesthetic with epinephrine infiltrated	86%
+30	Oxygenation restored with laryngeal mask airway and two-handed jaw thrust	99%
+32	Cricothyroidotomy incision made	-
+40	Cuffed 6.0 mm tracheal tube placed through cricothyroid membrane	95%
+47	Bilateral breath sounds confirmed	97%
+50	Transferred to operating room for attempted tracheostomy	-

**Figure 2:**
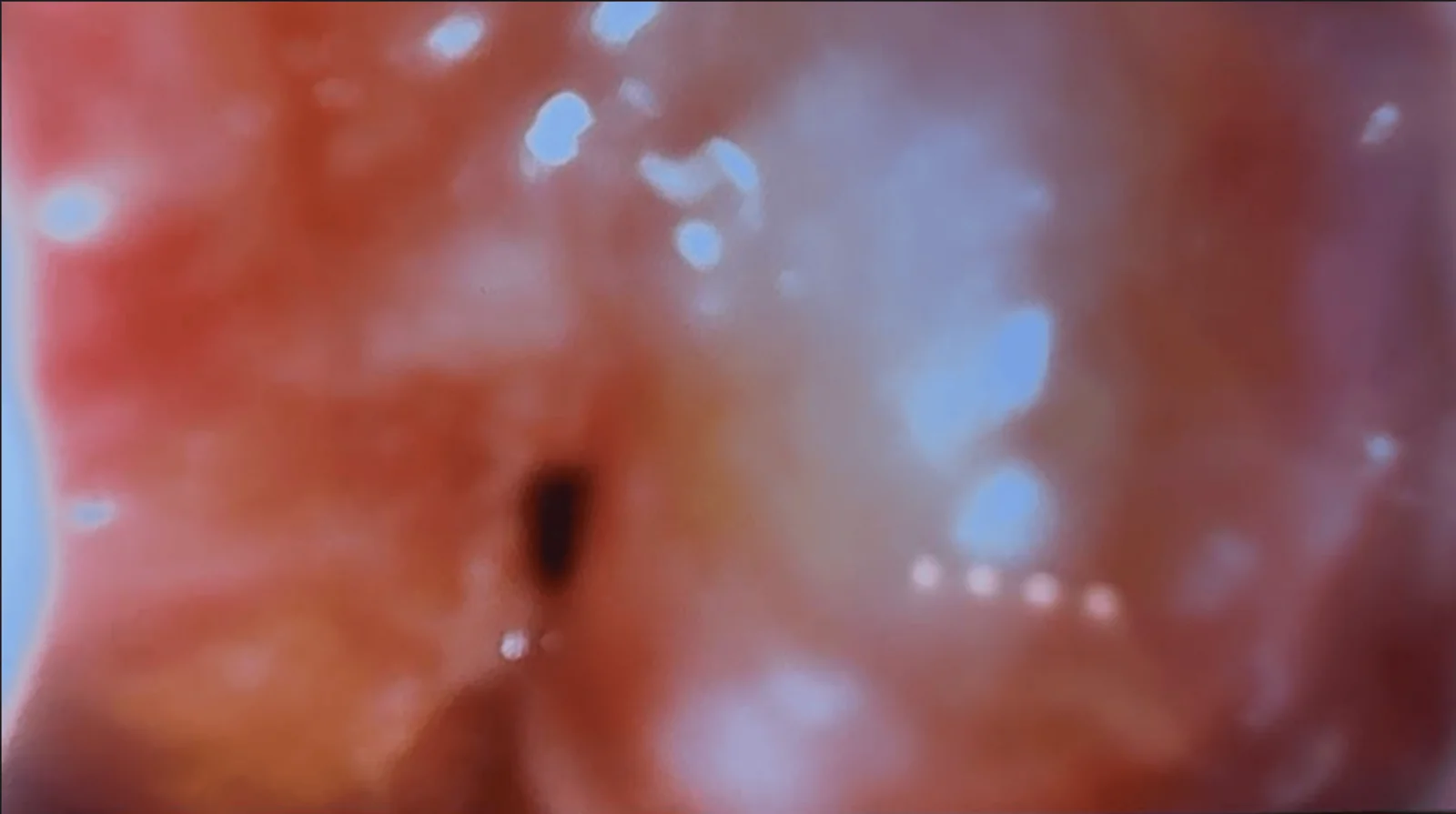
Fiberoptic laryngoscopy view of the epiglottis during attempted intubation Fiberoptic laryngoscopy view taken during the failed intubation attempt, showing erythematous and circumferentially edematous epiglottic mucosa. The remaining airway is a narrow slit, and the glottic inlet could not be traversed.

An anesthesiologist and a respiratory therapist arrived within four minutes. He was induced with ketamine to preserve spontaneous ventilation in two divided doses totaling 400 mg. No neuromuscular blocking agent was administered. Video laryngoscopy was attempted first. The epiglottis was too swollen to admit a tracheal tube, and saturation fell to 62%. Bag-mask ventilation brought it back to 90%. A flexible bronchoscope was attempted without success. Figure 2 shows the swollen epiglottis with small patency in the center at that point. General surgery was called. When saturation dropped again to 69% even with oral airway and bag-mask ventilation, a laryngeal mask airway was placed.

Manual ventilation through the laryngeal mask airway with a forceful two-handed jaw thrust maneuver at the same time restored oxygenation. The cricothyroidotomy began 27 minutes after the first laryngoscopy attempt, by which time saturation had come back to 99%. Local anesthetic with epinephrine was injected into the skin, and a general surgeon performed an open cricothyroidotomy at the bedside. A cuffed 6.0 mm tracheal tube was in place eight minutes after the incision, with bilateral breath sounds and a saturation of 97%. Forty minutes had passed since the decision to intubate. He was then taken to the operating room to convert to a formal tracheostomy, which was aborted as the trachea was too far from the skin for the procedure to be safe. The cricothyroidotomy tube was sutured to the skin, and he went to the intensive care unit sedated and paralyzed, initially on norepinephrine.

**Figure 3:**
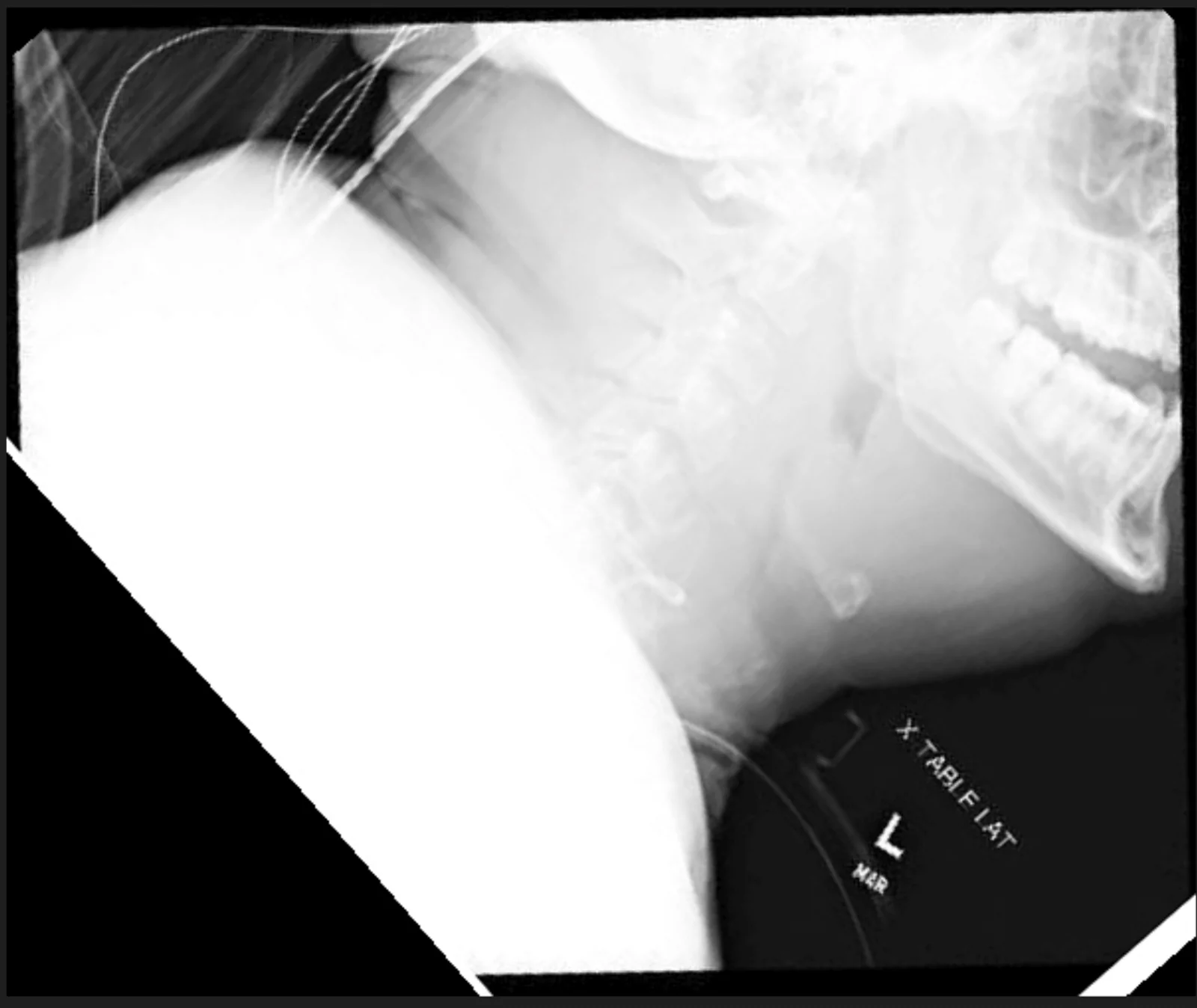
Cross-table lateral neck radiograph following cricothyroidotomy Cross-table lateral radiograph of the neck taken after emergency cricothyroidotomy, confirming the position of the cuffed tracheal tube through the cricothyroid membrane and showing the supraglottic airway still completely obstructed.

The submandibular swelling persisted on hospital day 2. He received intravenous methylprednisolone 40 mg every six hours, diphenhydramine, and ampicillin-sulbactam, with doxycycline added for suspected aspiration pneumonia. On hospital day 3, a chest radiograph showed the cricothyroidotomy tube in the right mainstem bronchus. The general surgeon repositioned it at the bedside, a repeat film confirmed placement, and saturation rose from 91% to 94%. A cross-table lateral neck radiograph the same day showed the tube in position and the supraglottic airway still completely obstructed (Figure 3). The white cell count rose to 26,000 per microliter.

On hospital day 4, he returned to the operating room for a planned intubation with an otolaryngologist present. Direct laryngoscopy succeeded, an epiglottic abscess was found and drained, and the cricothyroidotomy tube was exchanged for a 7.5 mm oral endotracheal tube advanced past the cricothyroidotomy site. Throat and abscess cultures both grew group G beta-hemolytic *Streptococcus*. Blood and respiratory cultures were negative.

His ventilator requirements climbed over the next day. By hospital day 5, he needed a fraction of inspired oxygen of 1.0, positive end-expiratory pressure of 20 cmH_2_O, a respiratory rate of 30, and a tidal volume of 430 mL, with a peak airway pressure of 30 cmH_2_O and a plateau pressure of 28 cmH_2_O. Arterial blood gas analysis showed a pH of 7.41, a carbon dioxide tension of 49 mmHg, and an oxygen tension of 68 mmHg, giving a ratio of arterial oxygen tension to inspired oxygen fraction of 68. The chest radiograph, clear on admission, now showed diffuse bilateral opacities. Severe acute respiratory distress syndrome was diagnosed, and he was transferred to a tertiary center for possible extracorporeal membrane oxygenation, which he did not end up needing. He also had rhabdomyolysis with a creatine kinase of 2,600 U/L and a triglyceride level of 431 mg/dL temporally associated with the propofol infusion.

At the tertiary center, he was sedated, paralyzed, and treated with corticosteroids, inhaled nitric oxide, and diuresis. Paralysis was stopped on hospital day 6 and nitric oxide on day 7. He received piperacillin-tazobactam, narrowed to ceftriaxone when cultures returned, broadened again for persistent fever, then narrowed back to ceftriaxone for a 14-day course on infectious diseases advice. Repeat computed tomography of the neck showed no residual abscess. Echocardiography on hospital day 6 showed moderate global left ventricular hypokinesis, severe right ventricular hypokinesis, and an ejection fraction of 35%, read as most consistent with stress cardiomyopathy. Guideline-directed medical therapy was started.

He was extubated to bilevel positive airway pressure on hospital day 11, moved to high-flow nasal cannula oxygenation, and was on low-flow nasal cannula by day 14. He reported left leg pain and had foot drop on examination, attributed clinically to a compressive peroneal neuropathy from prolonged positioning. At transfer, his left dorsiflexion was 1-/5, and plantar flexion was 2/5. Duplex ultrasonography done to investigate the leg pain showed acute deep vein thrombosis in the bilateral femoral and popliteal veins, which had formed despite prophylactic enoxaparin 40 mg twice daily. He was given a heparin infusion and changed to apixaban.

He was weaned to room air, finished his antibiotics without further fever or dyspnea, and went to acute inpatient rehabilitation on hospital day 24. He improved steadily there and went home with services on hospital day 46, walking with a rolling walker. The cricothyroidotomy stoma was still open and dressed at that point, with otolaryngology follow-up arranged and a repeat echocardiogram planned.

## Discussion

The most useful lesson here is what his response to treatment looked like. He was not a late presenter, and he was not dismissed. He sought care twice in one day and was examined both times carefully. The problem was that the treatment worked. Ninety minutes after epinephrine and dexamethasone, his stridor had settled, and he had gone from grunting to speaking in full sentences. Four hours later, he was breathing through a straw. Symptomatic improvement in supraglottic obstruction reflects a small change in mucosal edema at a steep point on the pressure-flow curve. It says nothing about whether the underlying infection has been controlled, and in this patient, a beta-hemolytic *Streptococcus* had already formed an abscess. Improvement should buy time for airway planning, not replace it. Taken together, progressive sore throat and dysphagia, a muffled voice, stridor, and a transient response to steroids and epinephrine should be read as a warning pattern for impending supraglottic obstruction rather than as a treated illness.

Two things about the initial workup made this harder. The first was the diagnosis. Angioedema is a fair reading of a swollen tongue, muffled voice, and stridor, and it prompts much the same immediate treatment. He was on amlodipine rather than an angiotensin-converting enzyme inhibitor, so the usual trigger was absent, and the anchor was the syndrome rather than any mechanism. The second was that his body defeated the imaging. The lateral neck film is the classic bedside test for epiglottitis, and the radiologist could report only submandibular edema before noting that extreme obesity had made the study uninterpretable. Computed tomography was ordered and refused, not out of preference but because he could not lie back on the scanner. Obesity limited the diagnostic pathway before it complicated the airway. When the airway is already threatened, and imaging is not going to answer the question, the better move is to go straight to a controlled airway assessment somewhere a surgical airway is immediately available [1,2,4].

His chart also held a warning that predated the illness. The home sleep study showed an apnea-hypopnea index of approximately 50, a nadir saturation in the mid-30s, and more than three hours below 89% in a single night. A pharynx that collapses 50 times an hour in health has nothing left to give when the supraglottis swells. Severe obstructive sleep apnea is worth treating as an acute airway risk factor, not just a chronic diagnosis on a problem list.

Once the airway was instrumented, the physiology behaved as expected. Low functional residual capacity and high oxygen consumption leave almost no reserve between attempts, and he desaturated to 62% on the first attempt and 69% on the second [5]. The same habitus that shortens apnea time also predicts difficult face mask ventilation, so the usual fallback is least reliable in exactly these patients [6]. The number of attempts available is set by the patient's physiology before anyone touches him, and it is not many.

The induction deserves comment. He was given ketamine in divided doses with no paralytic agent, which preserves spontaneous ventilation and airway tone and is a reasonable choice when obstruction is suspected. It is not the same as an awake technique with a topicalized airway and a bronchoscope as the primary plan, and the first device used was a video laryngoscope. The Difficult Airway Society 2025 guidelines keep the familiar sequence of tracheal intubation, supraglottic airway, facemask ventilation, and front-of-neck airway, but move the emphasis toward first-attempt success and toward moving through the algorithm without hesitating [8]. The Fourth National Audit Project is the reason that emphasis exists. Repeated attempts, failure to recognize the need for a surgical airway, and delay in performing one turned up again and again among deaths and hypoxic brain injuries, in operating rooms, emergency departments, and intensive care units alike [9,10]. American Society of Anesthesiologists guidance likewise advises limiting attempts and preparing invasive access alongside, rather than after, the other plans when difficulty is expected [11]. Epiglottitis plus extreme obesity meets those criteria before the patient is touched, and an awake bronchoscopy technique with the neck marked and a surgeon scrubbed is defensible as the first plan rather than the last.

What the team got right shows up in the timing. General surgery was called during the bronchoscopic attempt rather than after it, and the surgeon was at the bedside four minutes later. The incision was made at a saturation of 99%, not at the 69% nadir. It would be easy to conclude from this that the cricothyroidotomy was avoidable, since oxygenation had been restored. We think that reads it backwards. Ventilation was manual through a laryngeal mask airway, depended on one person maintaining a jaw thrust, and sat above an obstruction that was getting worse rather than better, and nothing non-surgical was left to try. Cutting then, with the patient oxygenated and the team assembled, is a different procedure from cutting four minutes later. The 2015 and 2025 Difficult Airway Society guidelines both treat scalpel cricothyroidotomy as a planned endpoint rather than a last resort, and the 2025 update standardizes a vertical-incision approach meant for necks where landmarks cannot be felt [8,12].

That standardization exists because the procedure is hardest in the patients who need it most. Identifying the cricothyroid membrane by inspection and palpation succeeds in 0% to 39% of obese subjects across published series [13]. The comparison cited earlier found 39% accuracy in obese versus 71% in non-obese patients, with neck circumference predicting failure [7]. Ultrasound raises the success rate substantially, and the 2025 guidelines endorse it for this purpose, though it takes longer than palpation and so belongs before airway management starts rather than during a crisis [8,13]. Our patient's procedure took eight minutes from incision to a secured tube, which is a long time for someone with his reserve. The practical conclusion is simple. In a patient with a large neck circumference or landmarks that cannot be palpated, find the cricothyroid membrane with ultrasound and mark it before the first laryngoscopy attempt.

The finding most worth reporting is that a bedside cricothyroidotomy succeeded while a tracheostomy could not be completed in the operating room, under controlled conditions, by the same service. The cricothyroid membrane sits close to the skin between two palpable cartilages. Below the cricoid, the trachea falls away, and a thick pretracheal fat pad and short neck make that distance longer still. Do not assume that a cricothyroidotomy in a patient of this size can be converted to a tracheostomy on the usual schedule. Plan for the possibility that the cricothyroidotomy tube is the medium-term airway.

Our patient showed what that means. On hospital day 3, the 6.0 mm tube ended up in the right mainstem bronchus and had to be repositioned. A short tube reaching the airway through a long tissue tract, in a neck that shifts with every transfer and every pull on the circuit, holds its position less well than a tracheostomy tube, and there is less distance to the carina to spare. This is a specific hazard of leaving a cricothyroidotomy in place in a large patient, and it is not much discussed. It argues for confirming position radiographically without much prompting, securing the tube carefully, and deciding at the time of insertion how and when the surgical airway will be exited [14]. Here, that plan was oral endotracheal intubation once the abscess had been drained, which worked on hospital day 4. The cricothyroidotomy stoma was nonetheless still open when he went home six weeks later, needing dressings and otolaryngology follow-up.

The organism and the abscess also shaped the airway course. Adult supraglottitis in the post-vaccination era is caused by a wider and less predictable range of organisms than the childhood disease it replaced, with beta-hemolytic streptococci among the more common isolates and no organism found in a large minority of cases [2]. Group G beta-hemolytic *Streptococcus* grew from both the throat and the abscess, and the presence of a drainable collection explains why three days of high-dose methylprednisolone did not open the airway. A supraglottis that has not improved after 48 to 72 hours of appropriate treatment should prompt a search for abscess, and formal staging of severity helps [15].

The rest of his course is a reminder that surviving the airway is not the same as surviving the illness. Severe acute respiratory distress syndrome by the Berlin definition, transfer for possible extracorporeal support, biventricular dysfunction with severe right ventricular hypokinesis, rhabdomyolysis, extensive proximal deep vein thromboses, and a peroneal neuropathy with lasting foot drop all followed a crisis that lasted 40 minutes [16]. Two of these were foreseeable. The thromboses developed on enoxaparin 40 mg twice daily, a fixed prophylactic dose in a man of more than 200 kg, which raises the question of weight-adjusted dosing or anti-Xa monitoring at this body mass. The neuropathy was attributed to immobility and paralysis in a patient whose weight makes turning and offloading genuinely hard, and it was the injury that limited his recovery most. He went home walking short distances with a walker, six weeks after a two-day sore throat.

This report has limits. It describes one patient retrospectively and supports no causal claims. The emergency department record is detailed but does not give the blade geometry of the video laryngoscope, the number of attempts with each device, the laryngeal view obtained, the operator's level of training, or the cricothyroidotomy technique beyond its being open and at the bedside, and it does not record whether ultrasound was used to find the cricothyroid membrane. Neck circumference was never measured, so the single measurement most predictive of difficult front-of-neck access is missing. We cannot say whether an awake fiberoptic bronchoscopic technique chosen earlier would have avoided the surgical airway. What the case does show is that in a man with a body mass index of 66.5 kg/m^2^ and a nearly obstructed supraglottis, cricothyroidotomy worked when video laryngoscopy, bronchoscopy, and a tracheostomy did not.

## Conclusions

Acute epiglottitis in a patient with super-super obesity brings together an obstructed airway, a patient with no physiological reserve, imaging limited by his body habitus, and a rescue procedure that the same body makes difficult. Here, a complete response to steroids and epinephrine preceded collapse by four hours; video laryngoscopy and bronchoscopy both failed, and a laryngeal mask airway restored oxygenation only with a two-handed jaw thrust, allowing a bedside cricothyroidotomy to be performed before saturation fell again. This provided successful emergency airway access where a tracheostomy could not be completed, though it did not constitute a definitive long-term airway. Treat symptomatic improvement in supraglottic obstruction as a pause rather than a trend. Read severe obstructive sleep apnea as evidence of an airway with nothing in reserve. In a patient with stridor who cannot lie flat, go to controlled airway management rather than to more imaging. In a patient with a large neck circumference or impalpable landmarks, find and mark the cricothyroid membrane before the first attempt. And treat a cricothyroidotomy tube as a temporary airway that will not hold itself in place, with a plan for leaving it made the moment it goes in.
